# Amyloid‐PET disclosure in Subjective Cognitive Decline: patient experiences and outcomes over time

**DOI:** 10.1002/alz.090651

**Published:** 2025-01-09

**Authors:** Heleen M.A. Hendriksen, Tanja J. de Rijke, Ellen M.A. Smets, Agnetha D. Fruijtier, Elsmarieke van de Giessen, Argonde C. van Harten, Mardou S. S. A. van Leeuwenstijn, Jetske van der Schaar, Calvin Trieu, Denise Visser, Leonie N.C. Visser, Wiesje M. van der Flier

**Affiliations:** ^1^ Alzheimer Center Amsterdam, Neurology, Vrije Universiteit Amsterdam, Amsterdam UMC location VUmc, Amsterdam Netherlands; ^2^ Amsterdam Neuroscience, Neurodegeneration, Amsterdam Netherlands; ^3^ Medical Psychology, Amsterdam UMC location AMC, University of Amsterdam, Amsterdam Netherlands; ^4^ Amsterdam Public Health, Quality of Care, Personalized Medicine, Amsterdam Netherlands; ^5^ Department of Radiology & Nuclear Medicine, Vrije Universiteit Amsterdam, Amsterdam UMC location VUmc, Amsterdam Netherlands; ^6^ Division of Clinical Geriatrics, Center for Alzheimer Research, Department of Neurobiology, Care Sciences and Society, Karolinska Institutet, Stockholm Sweden; ^7^ Epidemiology and Data Science, Vrije Universiteit Amsterdam, Amsterdam UMC location VUmc, Amsterdam, Noord‐Holland Netherlands

## Abstract

**Background:**

Communicating amyloid‐status and the subsequent risk of future cognitive decline to individuals with Subjective Cognitive Decline (SCD) is complex. Insight into how individuals with SCD evaluate the disclosure process and its impact could help optimize current practice. Therefore, the aim of our study was to examine experiences with, and outcomes of, amyloid‐PET disclosure in individuals with SCD over time.

**Method:**

Fifty‐seven participants from the Subjective Cognitive Impairment Cohort (SCIENCe) (66±8yrs, 21(37%)F, MMSE 29±1, 15(26%) amyloid positive (A+)) completed questionnaires one week prior (T0), one day after (T1) and six months after amyloid‐PET disclosure (T2). Questionnaires addressed patient‐reported experiences (satisfaction with the consultation, evaluation of the provided information, and decision regret) and outcomes (emotions and behavioral (intentions to) change). We analyzed open‐ended questions thematically and tested effects of amyloid‐status and/or time using Repeated Measures ANOVA, χ², Mann‐Whitney U, and Independent Samples t‐tests.

**Result:**

Independent of amyloid‐status, participants were satisfied with the consultation (scale 1‐10; 7.9±1.7) and the provided information (scale 1‐4; T1:3.3±0.9, T2:3.2±0.8). After six months, A+ participants reported significantly more information needs (45% vs. 12%, p=.02), e.g. about drug research and advice on what can be done to slow down disease progression. Decision regret was low, independent of amyloid‐status (scale 1‐5; A+:1.5±0.9, A‐:1.4±0.6, p=.525). Participants mentioned to appreciate information about their own health status and the possibility to act on this information. After six months, A+ participants more often reported to have recorded their wishes for the future (scale 1‐7; median(IQR): A+: 5(4‐7), A‐: 3(1‐4), p=.023). Negative emotions remained low overall over the six‐month period, independent of amyloid‐status (negative affect: F(2, 82) = 1.884, p=.158, p_interaction_=.997; uncertainty: F(2, 86) = 1.258, p=.289, p_interaction_=.618; anxiety: F(2, 80) = 1.014, p=.368; p_interaction_=.728).

**Conclusion:**

Participants with SCD evaluated amyloid‐PET disclosure positively, regardless of amyloid‐status. Amyloid‐PET disclosure did not induce negative emotions and participants did not regret learning their result. The reported need for information after six months, especially among A+ individuals, underscores the importance of follow‐up.

